# Constructing datasets to measure geographic variation in bereavement: integrating mortality, population structure, and survey-based probability

**DOI:** 10.3389/fpubh.2026.1809127

**Published:** 2026-07-03

**Authors:** Toni Miles, Ke Wang, Yanling Qi, Changle Li

**Affiliations:** 1 The Carter Center's Mental Health and Caregivers Program, Atlanta, GA, United States; 2 University of Georgia, College of Public Health, Athens, GA, United States; 3 Department of Health Care Management, College of Health and Human Services, California State University, Long Beach, Long Beach, CA, United States; 4 School of Health Management, Fujian Medical University, Fuzhou, China

**Keywords:** bereavement probability, bereavement surveillance, county-level estimation, data integration, household and family structure, mortality geography, grief

## Introduction

Geographic variation in mortality across the United States is substantial and well documented (1). Life expectancy, crude mortality rates, and cause-specific death rates vary across states, counties, and communities, reflecting differences in demographic composition, socioeconomic conditions, healthcare access, and local mortality environments (2, 3). Large-scale epidemiologic efforts, including national life expectancy analyses, U.S. Burden of Disease studies, and the Mortality Disparities in American Communities cohort, describe where and how deaths occur (4, 5). However, these systems quantify decedents, not the populations exposed to those deaths.

Mortality geography has advanced substantially, particularly through small-area analyses that identify where death rates are elevated, persistent, or changing over time. These studies are essential for understanding the distribution of mortality risk. However, they stop at the decedent. Bereavement surveillance requires a second step: translating death events into the populations and social units exposed to those deaths. The present Data Report therefore does not compete with mortality mapping. Rather, it extends the public health use of mortality data by linking observed deaths to survey-derived bereavement probability and household and family denominators.

Bereavement is a population-level exposure generated by mortality events (6). Each death can affect family members, household members, friends, caregivers, coworkers, and other members of a social network. Yet routine public health surveillance systems do not estimate the number or distribution of persons, households, or families exposed to death within defined geographic areas. Mortality rates therefore cannot serve as direct proxies for bereavement. They describe the occurrence of death, but not the populations living with its consequences.

The 2019 Georgia Behavioral Risk Factor Surveillance System bereavement module provided an empirical estimate of self-reported bereavement during a defined 24-month period (7). Li et al. reported this survey-derived probability for Georgia adults. The present Data Report makes a different contribution: it uses that probability as an empirical anchor for constructing geographically resolved bereavement datasets. Specifically, the report integrates BRFSS-derived bereavement probability with CDC WONDER county mortality data and American Community Survey household and family structure measures to generate county-level estimates under explicit calibration assumptions (8, 9).

This paper therefore does not attempt to identify the causes or health consequences of bereavement. Instead, it constructs a reproducible data infrastructure for translating individual-level bereavement probability into county-, household-, and family-level estimates. The purpose is to make assumptions visible, compare alternative calibration approaches, and demonstrate how publicly available data can support bereavement surveillance and planning in areas where bereavement is not directly measured.

## Data sources

Prior to merging, each data source was harmonized by year, state, county name, and 5-digit Federal Information Processing Standards (FIPS) code. The extracted data included population counts, resident deaths, and household and family composition for 2017 and 2018 in Georgia and Ohio. These years align with the 2019 Georgia BRFSS bereavement module item asking whether respondents had experienced the death of a family member or close friend during 2017–2018. The merged file was validated through missingness checks, outlier detection, and logical rule assessment. The 5-digit FIPS code was present in all datasets and uniquely identifies each county in the United States: the first two digits identify the state, and the last three identify the county (7, 10, 11). The publicly available data sources were:

### Behavioral risk factor surveillance system (BRFSS)

The 2019 Georgia Behavioral Risk Factor Surveillance System (BRFSS) state-added bereavement module provides the key empirical parameter used in this study: the proportion of adults reporting the death of a close family member or friend within the prior 24 months, corresponding to 2017–2018. (7) The analytic sample included 5,206 respondents from an initial sample of 7,354 respondents, representing 70.8% of the initial sample. The bereavement probability (*p* = 0.4538) was estimated using standard BRFSS survey weights (_LLCPWT), stratification (_STSTR), and primary sampling units (_PSU) to account for the complex survey design (9, 10).

Missing data were addressed using multiple imputation, with results combined across 50 imputed datasets to produce a single population-level estimate. This weighted, imputed estimate is used as the baseline probability in all calibration options. To assess the impact of survey design and missing data, the bereavement probability was also calculated using complete-case unweighted data. The relative difference between the unweighted and weighted estimates was 1.39%, indicating limited sensitivity to weighting and imputation. This parameter is treated as a population-level estimate of self-reported bereavement exposure within a defined 24-month period and serves as the empirical anchor for all subsequent scaling procedures.

The BRFSS design includes both bereaved and non-bereaved respondents within a representative population sample, allowing direct estimation of a population-level proportion.

### CDC wonder mortality files

To synchronize time across datasets, county-level resident deaths for 2017–2018 were extracted from CDC WONDER. These data were used to calculate county crude mortality rates and to characterize the mortality context in which bereavement exposure was modeled. In this framework, mortality data identify the occurrence and geographic distribution of deaths; they are not treated as direct measures of bereavement (4, 5).

### American community survey (ACS)

The American Community Survey (ACS) provides county-level household and family structure measures, including total households and total families. Additional ACS measures, such as household size and family size, can support future refinements of the framework. In this Data Report, ACS household and family counts are used as social-unit denominators for modeling the presence of bereavement within households and families under explicit calibration assumptions. A total of 247 counties in Georgia and Ohio were included (8).

## Dataset construction and workflow

**Figure 1:**
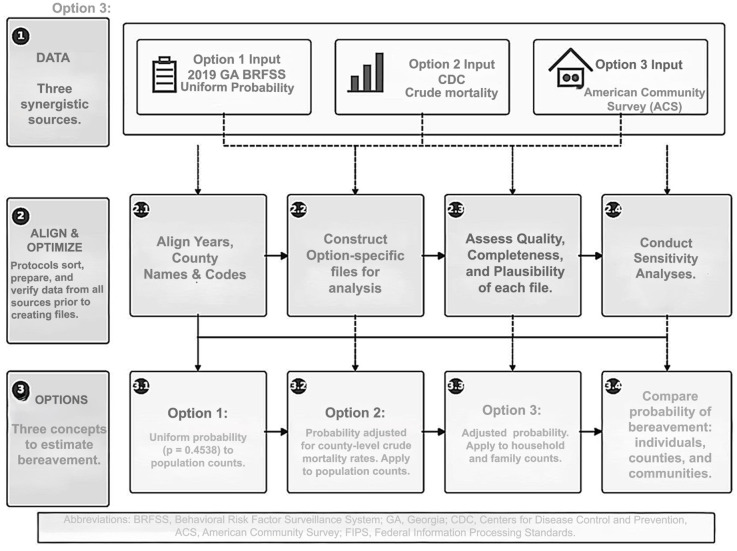
Workflow Final. BRFSS, Behavioral Risk Factor Surveillance System; GA, Georgia; CDC Wonder, Centers for Disease Control and Prevention; ACS, American Community Survey; FIPS, Federal Information Processing Standards.

Figure 1 illustrates the structured process used to construct a geographically resolved bereavement dataset for 247 counties in Georgia and Ohio. Three publicly available data sources are integrated: (1) the 2019 Georgia BRFSS, which provides a 24-month self-reported bereavement probability (*p* = 0.4538); (2) CDC WONDER mortality files, which provide county-level death counts used to calculate crude mortality rates; and (3) the ACS, which provides population, household, and family structure measures.

Data processing included harmonization of geographic identifiers, alignment of time periods to 2017–2018, and annualization of relevant multi-year aggregated values. Derived measures included crude mortality rates, standard errors calculated using the delta method under a Poisson assumption, and corresponding 95% confidence intervals. The dataset was evaluated using missing-value audits, logical consistency checks, and statistical outlier detection using the 1.5 × IQR rule (11).

Three calibration options were applied to translate individual-level bereavement probability into population-level estimates. Option 1 applies a uniform probability across all counties. Option 2 adjusts this probability using county-level crude mortality rates relative to the combined Georgia/Ohio reference crude mortality rate. Option 3 uses household and family structure to model bereavement exposure within ACS-defined social units.

The unit of observation in the final dataset is the county, with all measures aligned to a common time frame and standardized geographic identifiers. The dataset produces county-level estimates of bereaved individuals, households, and families under each calibration assumption, along with sensitivity analyses assessing robustness to variation in the baseline probability parameter.

### Structure of the final dataset / README section

File Name: merged_bereavement_GA_OH_2017_2018_ options1_3_WITH_QA.csv

Unit of Observation: County (5-digit FIPS), aggregated over 2017 - 2018 and annualized Geographic Coverage: 247 counties across Georgia (GA) and Ohio (OH)

Description: This dataset integrates mortality, population, household, and family structure data with a survey-derived bereavement probability to generate option-specific bereavement estimates under explicit calibration assumptions.

The dataset includes:

Geographic identifiers
Population and mortality measures
Derived mortality statistics (CMR, SE, 95% CI)
Household and family structure variables
Bereavement probability parameters (Options 1–3)
Option-specific bereavement estimates
Quality flags (statistical and logical checks)

## Dataset verification and quality assessment

The merged dataset underwent systematic screening for missing values, statistical outliers, and logical inconsistencies, including deaths exceeding population counts. These procedures were used to evaluate whether the merged file was suitable for deriving calibration variables and option-specific estimates (11).

### Missing-value audit

All required variables, including population, deaths, household counts, and family counts, were assessed for completeness. No missing values were identified in fields required for crude mortality rate calculations or calibration option development.

### Statistical outlier detection

The 1.5 × IQR rule was applied across 16 variables to identify statistical outliers. A total of 53 unique counties were flagged, with several counties identified across multiple variables. Review of these cases indicated that the flagged observations reflected true demographic extremes, including large metropolitan counties and sparsely populated rural counties. No observations were excluded on the basis of outlier status.

### Logical consistency checks

Each county was evaluated for demographic plausibility, including deaths exceeding population counts, implausible household or family sizes, and inconsistent rate calculations. No logical errors were identified. The dataset was therefore retained in full for calibration option development.

## Derived measures

After verification, derived mortality measures were calculated from the merged dataset and added to the analytic file. These measures included crude mortality rate (CMR) per 100,000 population, the standard error of the CMR using the delta method, and corresponding 95% confidence intervals. Although comparable measures are available through CDC WONDER, recalculating them from the merged file served as an internal quality-control step and confirmed consistency between source data and derived variables.

### Crude mortality rate (CMR)

Variation in CMR across counties reflects population age composition as well as cause-specific mortality patterns, healthcare access, socioeconomic conditions, environmental exposures, and injury burden. From a population-exposure perspective, this variation represents the local mortality environment. Counties with more deaths during a defined period create more opportunities for residents to encounter loss within their social networks. In this context, CMR is used as a contextual scaling variable reflecting mortality intensity, not as a measure of causal risk.

This application does not imply that demographic or environmental factors directly determine individual bereavement probability. Rather, the calibration options use CMR to represent differences in the number of deaths occurring within defined populations during the 24-month exposure window. Alternative specifications using age-standardized or age-specific mortality rates could be explored in future work. When constructing a dataset grounded in observed deaths within a defined period, CMR provides the most direct alignment with exposure occurrence.

Because CMR reflects deaths occurring within a defined time window, it can capture both chronic and acute shifts in mortality intensity within a community. Under the Option 2 calibration assumption, communities with higher CMR are assigned higher modeled bereavement probabilities. CMR is used rather than age-adjusted mortality rates because bereavement exposure arises from actual deaths, not from age-standardized mortality risk.

### Standard errors/confidence intervals

Standard errors were calculated using the delta method under the assumption that death counts follow a Poisson distribution. Corresponding 95% confidence intervals were calculated for each county-level CMR and used as descriptive measures of mortality-rate uncertainty. As shown in Tables 1 and 2, Georgia had a lower CMR than Ohio during 2017–2018.

**Table 1:** State-level crude mortality rates (CMR) per 100,000 (2017–2018).

State	CMR	95% CI lower	95% CI upper
Georgia	803.4	799.5	807.2
Ohio	1,061.8	1,057.6	1,066.0
Combined	939.6	936.7	942.5

CI, 95% Confidence interval.

**Table 2:** Sensitivity analysis, option 3 assumption, lower bound 40%, upper bound 50%.

Scenario	Median % change	Minimum % change	Maximum % change
Household (*p* = 0.40 vs. 0.4538)	−6.18%	−10.22%	−1.62%
Household (*p* = 0.50 vs. 0.4538)	+4.27%	+0.34%	+8.44%
Family (*p* = 0.40 vs. 0.4538)	−4.76%	−9.94%	−0.58%
Family (*p* = 0.50 vs. 0.4538)	+3.04%	+0.07%	+8.15%

*p*, probability of at least one person being bereaved in the prior 24 months.

### Calibration options

The 2019 Georgia BRFSS provides a probability of bereavement based on individual self-reports. Disparities in life expectancy and mortality are well described (1, 3, 4), but individual bereavement has not been routinely translated into estimates of geographically defined at-risk populations. The calibration options presented here move from individual bereavement probability to county-level bereavement estimates. They are not competing estimation techniques; rather, they formalize complementary questions about how bereavement may be scaled across geography and social units.

Option 1: Uniform probability
Option 2: Mortality-calibrated probability
Option 3: Social structure scaling

All three options use the baseline 24-month probability reported in the 2019 Georgia BRFSS bereavement module. The module asked respondents whether they had experienced the death of a family member or close friend during 2017–2018 (response options: Yes, No, Not sure, Refused). Respondents answering “Yes” were then asked how many losses occurred and to classify each decedent's relationship (e.g., spouse/partner, parent, sibling, child, other family member, friend, or neighbor). Responses of “Not sure” and “Refused” were excluded from the analytic denominator when calculating the bereavement probability. Although the module includes relationship categories and a defined 24-month window, it does not provide a formal operational definition of “close,” nor does it specify whether “experienced” refers to emotional impact, physical proximity, or awareness of a death within one's broader social network. Consequently, the baseline probability (*p* = 0.4538) reflects self-defined exposure to loss within a standardized time frame.

This feature is consistent with the BRFSS design as a population health surveillance system relying on self-report. A broader interpretation of “close” could increase the reported probability, while a narrower interpretation could reduce it; the direction and magnitude of this variability may differ across demographic or cultural contexts. Importantly, the BRFSS module includes both bereaved and non-bereaved respondents within a representative population sample. Many traditional bereavement studies enroll only bereaved individuals and do not include a population comparison group. The BRFSS design therefore provides a population-level proportion anchored to a defined denominator, making geographic scaling feasible. In this dataset, the probability parameter is treated as an empirically observed survey proportion rather than a clinically adjudicated measure of relational proximity, and all calibration options are explicitly anchored to this value.

This value represents the proportion of adults reporting the death of a close friend or family member in the 24 months prior to interview. This estimate represents approximately 3.7 million Georgia adults aged 18 years and older within an adult population of about 10 million persons (7). This value is used as Option 1—assumed uniform probability.

Options 2 and 3 incorporate additional assumptions about how local mortality intensity and social structure may influence modeled bereavement probabilities. Option 2 uses an indirect calibration approach, drawing on the logic of indirect rate standardization (IRS), to scale the BRFSS probability by county CMR relative to a reference CMR (12, 13). Because small numbers can produce unstable rates, we also examined the data for potential small-number instability using the enhanced modified Kalman filter framework (12, 13). Quality assessment showed that no county had fewer than 20 deaths in either year; therefore, the traditional IRS-based calibration was retained.

#### Option 1—uniform Georgia 2019 BRFSS probability (Popt1)

Option 1 provides a neutral baseline. It assumes a uniform distribution of bereavement likelihood and is derived from the weighted 2019 Georgia BRFSS state-optional bereavement module (7). All geographic units are assigned the same probability during the 24-month pre-survey period. Under this option, variation in the estimated number of bereaved persons is driven by resident population size. The at-risk population is defined by the analytic question. To estimate the number bereaved for a geographic unit as shown in Equation 1:


$$\begin{array}{l} P\;opt\;1=\;Prevalence\;\left(weighted\right)=p\;opt\;1=0.4538 \end{array}$$ (1)

#### Option 2—mortality-calibrated bereavement probability (Popt2)

Option 2 should be read as a bridge between mortality geography and bereavement geography: county mortality intensity modifies the survey-derived bereavement probability, but the resulting value remains a modeled bereavement exposure estimate rather than an observed mortality rate as shown in Equation 2.

Option 2 adjusts the BRFSS-derived bereavement probability by county crude mortality intensity. The purpose is not to infer bereavement directly from mortality rates, but to ask how the survey-derived probability changes when counties differ in the density of deaths occurring during the same exposure window. The county CMR is therefore used as a contextual calibration factor.

For each county, the Option 2 probability is calculated as:


$$\begin{array}{l} P\;opt2=P\;opt1\;\times \left(CMR\;county\;/\;CMR\;ref\right) \end{array}$$ (2)

where Popt1 = 0.4538, CMR_county_ is the county crude mortality rate, and CMR_ref_ is the combined Georgia/Ohio reference crude mortality rate of 939.6 deaths per 100,000 population. Values are expressed as probabilities and can also be presented as estimated bereavement rates per 100,000 population.

This calibration preserves the BRFSS bereavement probability as the empirical anchor while allowing county-level mortality environments to modify the estimate. Counties with crude mortality rates below the reference value receive lower Option 2 probabilities; counties with crude mortality rates above the reference value receive higher Option 2 probabilities. Thus, Option 2 does not replace the survey estimate. It redistributes that estimate according to observed county-level mortality intensity.

The formula could theoretically produce probabilities greater than 1.0 in extremely high-mortality settings. For this reason, calculated probabilities were bounded to the interval [0,1]. In the present Georgia/Ohio dataset, no county exceeded the upper bound; therefore, no truncation was applied.

To illustrate the calibration logic, we compare two Georgia counties: Gwinnett and Quitman. Gwinnett County had a much larger annualized population than Quitman County, but a substantially lower crude mortality rate. Under Option 1, the same bereavement probability is applied to both counties, so the estimated number of bereaved persons is driven primarily by population size. Under Option 2, lower mortality intensity in Gwinnett reduces the adjusted bereavement probability, while higher mortality intensity in Quitman increases it. This comparison demonstrates the utility of Option 2: it makes visible how counties can differ not only in population size, but also in the mortality context in which bereavement occurs.

The American Community Survey allows analysts to distinguish between households and families as social units. As defined by ACS, a household includes related (kin) and unrelated (non-kin) individuals living in the same housing unit. A family is limited to persons living in the same unit who are related by birth, marriage, or adoption. This distinction advances bereavement accounting in two ways. First, it locates a bereaved individual within a residence. Second, it differentiates whether co-resident others are kin or non-kin. This approach moves beyond prior research that treats bereaved individuals as singular units. Although not shown in this Data Report, ACS provides additional detail on relationships among household residents.

#### Option 3—social structure scaling

Option 3 extends the individual-level bereavement probability to ACS-defined social units by estimating the modeled probability that a household or family contains at least one bereaved member during the defined 24-month period. This approach uses household and family structure to translate individual-level exposure into social-unit exposure under explicit calibration assumptions.

This formulation does not estimate the number of bereaved individuals within a household or family, nor does it account for clustering of bereavement within units. Instead, it defines a structural exposure indicator, identifying social units where bereavement is present under the assumption that exposure is distributed across the population. Option 3 should therefore be interpreted as a scaling mechanism linking individual exposure to social units, rather than as a direct estimate of within-household bereavement dynamics.

## Interpretation across options

Together, the three options provide a flexible framework for assessing bereavement extent across geographic areas and social units.

Option 1 offers a baseline for comparison and assumes uniform probability.
Option 2 redistributes bereavement estimates according to mortality differences across counties.
Option 3 links geography, mortality, and population social structure using explicit calibration assumptions.

**Figure 2:**
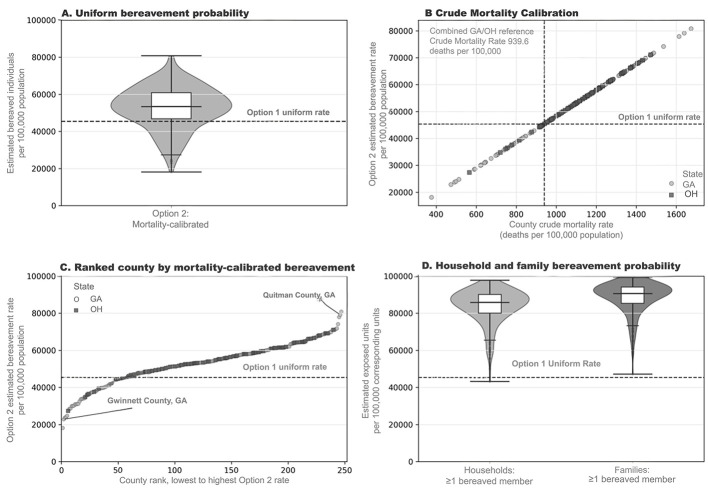
County bereavement estimates options1 3 GWINNETT QUITMAN. **(A)** Individual bereavement rate distribution. **(B)** Mortality calibration of individual probability. **(C)** Ranked county variation in mortality-calibrated estimates. **(D)** Household and family exposure distributions. Estimates are based on 247 counties in Georgia and Ohio. Option 1 applies the uniform 2019 GA BRFSS bereavement probability (*p* = 0.4538). Option 2 calibrates individual probability using county crude mortality relative to the combined GA/OH reference CMR. Option 3 estimates household and family exposure as the probability that at least one member is bereaved, using mortality-calibrated probability and ACS average household/family size. ACS household and family measures are 2018 ACS 5-year period estimates and were not annualized.

Figure 2 is designed to separate three quantities that are often conflated: the occurrence of deaths, the modeled probability of bereavement exposure among individuals, and the modeled presence of bereavement within households or families. The panels use mortality as context, not as a substitute for bereavement.

Figure 2 summarizes county-level bereavement estimates across the three calibration approaches. The 247 counties are treated as a single sample in Panel A, which shows the distribution of mortality-calibrated individual bereavement rates across Georgia and Ohio. The dashed line marks the uniform Option 1 rate derived from the 2019 Georgia BRFSS probability. Panel B shows the linear relation between county CMR and Option 2 mortality-calibrated bereavement rates. The vertical dashed line identifies the combined Georgia/Ohio reference CMR used for calibration. Panel C ranks counties from lowest to highest Option 2 rate, illustrating how mortality calibration redistributes estimates across counties while preserving geographic heterogeneity. Panel D extends the calibration to ACS household and family units, showing the distribution of units classified as having at least one bereaved member under the social-structure scaling approach.

Together, the panels show that the three options are not competing claims about a single true value. They are structured assumptions for translating a survey-derived individual probability into geographic and social-unit estimates.

### Panel A—individual bereavement rate distribution

Figure 2A shows the distribution of bereavement rates calculated under the Option 1 and Option 2 assumptions. The dashed horizontal line shows that, under the uniform probability assumption, the Option 1 rate is 45,380 bereaved persons per 100,000 population.

The box plot and violin graph show the distribution of county-level Option 2 bereavement rates per 100,000 population. Option 2 applies the formula 0.4538 × (CMRcounty / CMRref), where the combined Georgia/Ohio reference CMR is 939.6 deaths per 100,000 population. Counties with crude mortality rates below the reference CMR fall below the Option 1 line, while counties with crude mortality rates above the reference CMR fall above it. The violin plot highlights the distribution of mortality-calibrated estimates across counties and shows that many counties have modeled Option 2 rates above the uniform Option 1 rate. These values should be interpreted as modeled rates under the mortality-calibration assumption, not as directly observed county bereavement rates.

### Panel B—mortality calibration of individual probability

Panel B plots county CMR per 100,000 population on the x-axis against Option 2 mortality-calibrated bereavement rates per 100,000 population on the y-axis. No county had fewer than 20 deaths in either year, reducing concern that modeled Option 2 rates were driven by very small death counts. This panel shows how county-level mortality intensity changes the modeled bereavement probability under the Option 2 calibration assumption.

### Panel C—ranked county variation in mortality-calibrated bereavement rates

To provide a readable summary of mortality-calibrated bereavement rates, Panel C ranks the 247 counties by their adjusted rates. The lighter circles are Georgia counties. Consistent with Panel A, Panel C shows that, after adjustment for crude mortality, more than 200 counties have modeled rates greater than would be estimated using a uniform probability alone.

The utility of the calibration process can be illustrated by comparing Gwinnett and Quitman counties in Georgia. The two counties differ substantially in population size and mortality context. Gwinnett County had an annualized population of 924,020.5 persons and a crude mortality rate of 472.7 deaths per 100,000 population. Quitman County had an annualized population of 2,318.5 persons and a crude mortality rate of 1,639.0 deaths per 100,000 population. Gwinnett and Quitman illustrate opposite ends of the calibration problem: one county has a large population with lower mortality intensity, while the other has a small population with high mortality intensity.

Under Option 1, the uniform BRFSS probability is applied to both counties. This produces an estimated 419,321 bereaved persons in Gwinnett and 1,052 in Quitman. Under Option 2, the probability is calibrated to county CMR. The adjusted probability decreases to 0.2283 in Gwinnett and increases to 0.7916 in Quitman. Thus, Gwinnett remains the county with the larger estimated number of bereaved persons because of its population size, but Quitman has a much higher mortality-calibrated probability of bereavement exposure.

When the same logic is extended to families under Option 3, the modeled family-level probability is 0.6077 in Gwinnett and 0.9925 in Quitman. These estimates should be interpreted as the modeled probability that a family unit contains at least one bereaved member under the calibration assumptions, not as a direct count of bereaved individuals within families. The comparison shows why calibration is useful: it distinguishes population volume from mortality intensity and allows both to inform geographic estimates of bereavement exposure. Household-level estimates capture broader social exposure, including non-kin cohabitants; family-level estimates approximate exposure within kin-based residential units.

This example also illustrates a potential planning application. A death within a household or family can alter caregiving roles and related responsibilities, especially when the deceased person was a caregiver or care recipient. The present dataset does not measure caregiving redistribution directly, but ACS household and family denominators could support future analyses of how modeled bereavement exposure intersects with co-resident caregiving structures.

### Panel D—household and family exposure distribution

Panel D extends the mortality-calibrated probability to ACS household and family units. Both distributions are shifted well above the Option 1 individual probability line, indicating that when bereavement probability is scaled to co-resident social units, a large share of households and families are classified as containing at least one bereaved member. The family distribution is modestly higher than the household distribution, consistent with the model's use of family structure to estimate the probability that at least one kin member is bereaved. These estimates should be interpreted as modeled exposure of social units, not as counts of bereaved individuals within those units.

Households can include non-kin residents, while families are limited to kin. Households and families show a similar pattern of modeled bereavement burden, with rates clustered above 80,000 per 100,000 units. The modeled distribution of exposed social units—households and families classified as having at least one bereaved member—is calculated under the Option 3 assumptions.

When the mortality-calibrated individual probability is translated to ACS household and family units, both types of social units show high estimated exposure to bereavement. The family distribution is shifted somewhat higher than the household distribution, indicating that kin-based family units have a higher modeled probability of containing at least one bereaved member than household units under the calibration assumptions.

## Dataset application

### Sensitivity analyses

The BRFSS bereavement probability estimate (*p* = 0.4538) was derived from a single state dataset, Georgia. This sensitivity analysis addresses uncertainty in the probability value but not uncertainty in its transferability to another state. A one-way sensitivity analysis was conducted to assess the robustness of estimates across a plausible range of values. Two alternative probabilities were evaluated:

Lower bound: *p* = 0.40; upper bound: *p* = 0.50. These values reflect potential variability in the self-reported probability of bereavement based on published literature and survey design considerations. This sensitivity analysis uses the social-structure scaling approach (Option 3), which adjusts for crude mortality intensity and household or family structure. The table shows the percent change in bereavement rate when the BRFSS-derived uniform probability varies from 0.4000 to 0.5000.

Varying the input bereavement probability between 0.40 and 0.50 produced adjusted percent changes of approximately ± 5% to ± 10%. This pattern was similar for households and families. Although not shown, a similar county-level sensitivity approach showed that geographic rankings remained stable.

These findings suggest that Option 3 outputs are not highly sensitive to modest variation in the baseline probability parameter. This degree of tolerance is particularly important for counties with small populations. The sensitivity analysis further supports the use of BRFSS-based bereavement proportions as a practical foundation for county-level bereavement estimation.

## Dataset limitations and value

This Data Report addresses a gap left by mortality surveillance. Existing mortality systems identify decedents and increasingly allow detailed geographic analysis of death rates, trajectories, and risk environments. They do not, however, estimate the population living with the consequences of those deaths. By integrating mortality counts, BRFSS-derived bereavement probability, and ACS household and family denominators, this dataset creates an infrastructure for modeling bereavement exposure across geographic and social units.

This dataset was constructed to move individual bereavement probability to larger geographic and social units. The approach is promising, but several limitations should guide interpretation and future development.

The 24-month bereavement probability (*p* = 0.4538) is derived from a single state, Georgia, at a single time point. No comparable bereavement module was administered in Ohio. Applying this probability to Ohio therefore assumes transferability of self-reported exposure across states. Georgia and Ohio differ in factors that influence mortality rates. During the study period, Ohio's CMR (1,061.8 per 100,000) was higher than Georgia's CMR (803.4 per 100,000), a difference that could influence the true proportion of adults reporting bereavement. The sensitivity analysis (*p* = 0.40 to 0.50) evaluates the robustness of option-based outputs to variation in the probability parameter, but it does not establish that Ohio's true value lies within this range. For this reason, the numerical estimates presented for Ohio should be interpreted as option-based projections under an explicit assumption of parameter transferability.

The inclusion of Ohio serves two purposes: to demonstrate how the calibration framework performs under a different mortality environment, and to illustrate how publicly available mortality and demographic data can be integrated in a state lacking a bereavement surveillance module. The methodological framework is generalizable to any geographic area where mortality counts and household structure data are available. However, state-specific bereavement probability estimates would strengthen external validity. Expansion of BRFSS bereavement modules to additional states, or incorporation of national survey items with comparable time framing, would allow refinement of the probability parameter in future iterations of this dataset.

The framework can be extended nationally and adapted to new BRFSS modules, mortality data releases, or demographic indicators. It can also be adapted to other settings where mortality counts and household or family denominators are available, provided that local or transferable bereavement probability estimates are clearly specified.

CMR is contextual and dynamic. This dynamism is most visible when mortality data are examined over time. In this framework, CMR provides the temporal and geographic context needed to consider future public health action addressing the downstream effects of bereavement in small geographic areas.

The constructed dataset provides a flexible, reproducible foundation for quantifying bereavement burden beyond the individual level. By combining mortality statistics, household structure data, and BRFSS-derived probability, the framework offers a novel approach to understanding the force of mortality as it may be distributed through communities. Each death generates social, familial, and household consequences that cannot be inferred from mortality rates adjusted for population age or specific causes. The 2019 Georgia BRFSS module took an important step by measuring bereavement during a defined 24-month period and identifying a population-level probability of approximately 45%. Multi-state assessment of bereavement would further strengthen the generalizability of this estimate. The 2022 General Social Survey also contains a national item querying the death of someone close in the past 5 years and may provide an additional source for future estimation of bereavement proportions.

Option 3 uses American Community Survey (ACS) data describing the number of households and families in which bereavement could be present (i.e., at least one member is bereaved) during the specified 24-month window. In the ACS, households represent residential units that may include kin and non-kin residents, whereas families capture kin-based units within households. The framework does not estimate the probability that multiple individuals within the same household or family are bereaved, nor does it infer the number of bereaved individuals residing within a given unit. Instead, household- and family-level quantities are constructed as structural denominators for identifying units where bereavement is present under explicit calibration assumptions. In settings where deaths cluster within kin networks, multiple bereaved individuals may reside in the same household; this affects exposure intensity within the unit but does not change whether the unit is classified as bereaved. If the analytic goal shifts from identifying affected units to estimating numbers of bereaved individuals, within-household dependence and clustering would become more consequential. Future extensions could incorporate multilevel or simulation approaches to better represent clustering and intensity of exposure within households and families.

Differences observed across the three options reflect alternative calibration assumptions rather than differences in statistical accuracy. Option 1 applies a uniform survey-derived probability. Option 2 adjusts that probability to reflect contextual mortality intensity. Neither option is inherently “correct”; each is designed to address a distinct analytic question. Option 2 asks: What would county-level bereavement look like if the survey-derived probability were adjusted for county CMR? Option 3 asks: What would the social structure of the county—households or families—look like after accounting for differences in mortality context? The choice among options depends on the planning objective. A uniform framework may be useful for establishing a baseline awareness estimate, whereas a mortality-calibrated framework may be appropriate when contextual mortality differences are relevant for resource planning. The dataset is constructed to allow transparent comparison across these assumptions.

The household and family estimates are derived from ACS data. The sensitivity analyses assume independence of bereaved individuals within a household or family, but social networks and kin structures may not follow independence assumptions. This has implications for clinical and public health professionals seeking to address gaps created by deaths in a community. ACS household and family estimates also have margins of error (MOE). This source of uncertainty is not currently incorporated into the calibration options and represents an area for future methodological refinement.

CMR reflects population age structure: older populations generally have higher mortality rates. Bereavement, however, can occur at any age. The use of CMR is therefore a feature rather than a flaw in this application because it captures mortality intensity within a defined time frame. This feature should be noted when interpreting estimates.

Across the three datasets, some variables were conceptually similar but not identical, reflecting differences in survey design and administrative purpose. Harmonization therefore required aggregation and standardization, which may reduce granularity but improves comparability. These differences were considered during interpretation and are not expected to materially alter the application of the method.

This Data Report proposes a foundation for estimating bereavement geography. Mortality geography identifies where deaths occur; bereavement geography asks where the consequences of those deaths may be distributed across persons, households, families, and counties. As such, this work represents a beginning for this line of research.

### Dataset value

The use of crude mortality rates ensures that calibration parameters reflect the actual number of deaths occurring in a community. CMR is appropriate for this application because bereavement exposure arises from real deaths rather than age-standardized patterns. CMR is derived from death certificates and is integrated into multiple public data sources, supporting replication and extension.

The dataset relies entirely on publicly accessible, nationally maintained data systems: BRFSS, CDC WONDER, and ACS. This makes the approach reproducible and adaptable to other geographic regions where mortality is measured and households or families are counted. Any state or locality could strengthen the framework by adding a bereavement module that ascertains loss during a defined time window.

Numbers of documented deaths and numbers of households/families are already collected by most governmental surveys and linked to county/state. This approach supports replication of this work allowing extension to other states.

Household and family-level exposure capture different mechanisms. Households flag the presence of bereavement in residential units that may include kin and non-kin cohabitants. Families flag exposure within kin-based units, including spouses, children, and other co-resident relatives. These distinctions support planning questions that extend beyond individual bereavement.

The calibration options provide analytic flexibility. They allow users to compare how different assumptions affect county-level bereavement estimates and to select the option that best fits a research or planning question. In this dataset, outlier detection confirmed that apparent extremes reflected meaningful population heterogeneity, including large urban counties, small rural counties, and counties with unusually high or low mortality. Recognizing these differences is essential for accurate bereavement estimation and planning.

In this dataset, deaths are not the endpoint; they are the initiating event. By modeling population-level bereavement probabilities, the dataset may support future planning efforts, particularly when paired with local validation data. Because the health effects of grief are often framed primarily around prolonged grief disorder (14, 15), population-level estimates may help identify geographic areas where follow-up research or service planning should be prioritized.

Together, these features make the dataset a practical infrastructure for moving bereavement from an individual experience to a measurable public health exposure across geography and social structure.

### Data availability and Code availability

All data in this study were accessed from publicly available sources:

**Behavioral Risk Factor Surveillance System (BRFSS)**, Centers for Disease Control and Prevention, https://www.cdc.gov/brfss/.

**CDC WONDER Mortality Files**, Centers for Disease Control and Prevention https://wonder.cdc.gov/ [January 11, 2026].

**American Community Survey (ACS)**, United States Census Bureau https://www.census.gov/programs-surveys/acs/ [January 11, 2026].

Supplementary materials include the merged analytic data set created for this study, along with a full variable dictionary and README documentation.

## Data Availability

The original contributions presented in the study are included in the article/Supplementary material, further inquiries can be directed to the corresponding author.

## Supplementary material

Supplement 1 README MATCHED 2017–2018.

Supplement 2 Merged bereavement GA OH 2017–2018 options1 3 WITH QA.

Supplement 3 QA flagged counties merged bereavement GA OH 2017–2018.

Supplement 4 QA statistical outlier variable frequency may 2.
