# Supplementary material for: Constructing datasets to measure geographic variation in bereavement: integrating mortality, population structure, and survey-based probability
Source: Front Public Health. 2026 Jul 3;14:1809127. doi: 10.3389/fpubh.2026.1809127 (PMC13378018; doi:10.3389/fpubh.2026.1809127)
Supplement: Supplement 1 — README MATCHED 2017–2018. [file Data_Sheet_1.pdf]

# README – Supplement 1

*County-Level Bereavement Exposure Dataset, Georgia and Ohio, 2017–2018*

## File Overview

| Field | Description |
|---|---|
| **File name** | merged_bereavement_GA_OH_2017_2018_options1_3_WITH_QA.csv |
| **Unit of observation** | County (5-digit FIPS), with mortality aggregated over 2017–2018 and annualized counts included. |
| **Geographic coverage** | 247 counties across Georgia (GA; n = 159) and Ohio (OH; n = 88). |
| **Purpose** | This dataset integrates county-level mortality, population, and household/family structure data with a survey-derived bereavement probability to generate modeled estimates of bereavement exposure under three calibration approaches. |
| **Source alignment** | The 2017–2018 mortality window is aligned with the 24-month lookback period of the 2019 GA BRFSS bereavement module. |

## Contents

- Geographic identifiers
- Population and mortality measures
- Derived mortality statistics (CMR, SE, 95% CI)
- ACS household and family structure variables
- Bereavement probability parameters for Options 1–3
- Modeled bereavement estimates for individuals, households, and families
- Quality assessment flags for statistical and logical review

## Derivation Rules

| Measure | Derivation / Definition |
|---|---|
| **Annualized population** | pop_ann = pop_2yr / 2 |
| **Annualized deaths** | deaths_ann = deaths_2yr / 2 |
| **Crude mortality rate** | cmr_ann = (deaths_2yr / pop_2yr) × 100,000. This is numerically equivalent to (deaths_ann / pop_ann) × 100,000 because both numerator and denominator are divided by 2. |
| **CMR standard error and confidence interval** | cmr_se, cmr_ci_lower, and cmr_ci_upper are retained from the CDC WONDER county-level mortality output and are based on the 2017–2018 aggregate mortality file. |
| **Reference CMR** | cmr_ref = combined Georgia + Ohio CMR for 2017–2018 = 939.596 deaths per 100,000 population. |
| **Option 1 – Uniform probability** | brfss_prev_opt1 = 0.4538; bereaved_est_opt1 = pop_ann × brfss_prev_opt1. |
| **Option 2 – Mortality-calibrated** | cmr_ratio_ref = cmr_ann / cmr_ref; brfss_prev_opt2_raw = brfss_prev_opt1 × cmr_ratio_ref; brfss_prev_opt2 is bounded to [0,1]; bereaved_est_opt2 = pop_ann × brfss_prev_opt2. |
| **Option 3 – Social structure: households and families** | Option 3 translates the county-specific mortality-calibrated probability into unit-level exposure. hh_prev_opt3 = 1 − (1 − brfss_prev_opt2)^hh_size_avg; bereaved_hh_opt3 = hh_total × hh_prev_opt3. fam_prev_opt3 = 1 − (1 − brfss_prev_opt2)^fam_size_avg; bereaved_fam_opt3 = fam_total × fam_prev_opt3. |

## Key Assumptions

- Bereavement probability is based on the 2019 GA BRFSS bereavement module and represents self-reported bereavement within the prior 24 months.
- The Georgia baseline probability is transferred to all counties in Georgia and Ohio as a scenario-based parameter.
- Mortality provides contextual exposure intensity and is used to calibrate county-level probability in Option 2.
- Option 3 treats a household or family as bereaved if at least one member is estimated to have experienced bereavement.
- Household and family calculations assume independence of bereavement exposure within units.

- ACS margins of error are retained but not propagated into model uncertainty.
- Modeled outputs are scenario-based estimates, not directly observed counts of bereaved individuals, households, or families.

## Quality Assessment Results Included in Dataset

- The merged file contains 247 counties: 159 Georgia counties and 88 Ohio counties.
- No missing values were identified in required calculation fields. Blank values in statistical_outlier_vars occur only when statistical_outlier_flag = FALSE.
- The 1.5×IQR statistical outlier screen flagged 53 counties. These flags identify counties requiring review; they are not exclusion criteria.
- Logical consistency checks identified 0 counties with logical errors.
- All counties were retained in the analytic dataset.

## Variable Dictionary

| Variable | Domain | Description | Type |
|---|---|---|---|
| state_fips | Geographic identifiers | Two-digit state FIPS code. Georgia = 13; Ohio = 39. | Integer |
| state_abbr | Geographic identifiers | State postal abbreviation. | String |
| county_fips | Geographic identifiers | Five-digit county FIPS code; first two digits identify the state. | Integer |
| county_name | Geographic identifiers | Standardized county plus state label. | String |
| pop_2yr | Population and mortality | Total population denominator from the CDC WONDER 2017–2018 mortality file. | Numeric, count |
| deaths_2yr | Population and mortality | Total deaths from the CDC WONDER 2017–2018 mortality file. | Numeric, count |
| pop_ann | Population and mortality | Annualized population denominator calculated as pop_2yr / 2. | Numeric, count |
| deaths_ann | Population and mortality | Annualized deaths calculated as deaths_2yr / 2. | Numeric, count |
| cmr_ann | Mortality measures | Crude mortality rate per 100,000 population for the 2017–2018 aggregate file. Numerically equivalent to deaths_ann / pop_ann × 100,000. | Numeric per 100,000 |
| cmr_se | Mortality measures | Standard error for CMR as reported in CDC WONDER for the 2017–2018 aggregate mortality file. | Numeric per 100,000 |
| cmr_ci_lower | Mortality measures | Lower 95% confidence interval for CMR as reported in CDC WONDER. | Numeric per 100,000 |
| cmr_ci_upper | Mortality measures | Upper 95% confidence interval for CMR as reported in CDC WONDER. | Numeric per 100,000 |
| hh_total | ACS household/family structure | Total households from ACS S1101 2018 5-year file. | Numeric, count |
| hh_moe | ACS household/family structure | Margin of error for total households. | Numeric, count |
| hh_size_avg | ACS household/family structure | Average household size. | Numeric |
| hh_size_moe | ACS household/family structure | Margin of error for average household size. | Numeric |
| fam_total | ACS household/family structure | Total families from ACS S1101 2018 5-year file. | Numeric, count |
| fam_moe | ACS household/family structure | Margin of error for total families. | Numeric, count |
| fam_size_avg | ACS household/family structure | Average family size. | Numeric |
| fam_size_moe | ACS household/family structure | Margin of error for average family size. | Numeric |
| brfss_prev_opt1 | Option 1 – Uniform probability | Baseline 24-month bereavement probability from the 2019 GA BRFSS bereavement module; p = 0.4538. | Numeric probability |
| bereaved_est_opt1 | Option 1 – Uniform probability | Estimated bereaved individuals under uniform probability: pop_ann × brfss_prev_opt1. | Numeric, count |

| | | | |
|---|---|---|---|
| cmr_ref | Option 2 – Mortality-calibrated | Combined Georgia + Ohio 2017–2018 reference CMR = 939.596 deaths per 100,000 population. | Numeric per 100,000 |
| cmr_ratio_ref | Option 2 – Mortality-calibrated | County CMR divided by reference CMR: cmr_ann / cmr_ref. | Numeric ratio |
| brfss_prev_opt2_raw | Option 2 – Mortality-calibrated | Unbounded mortality-calibrated probability: brfss_prev_opt1 × cmr_ratio_ref. | Numeric probability |
| brfss_prev_opt2 | Option 2 – Mortality-calibrated | Bounded mortality-calibrated probability constrained to [0,1]. | Numeric probability |
| bereaved_est_opt2 | Option 2 – Mortality-calibrated | Estimated bereaved individuals under mortality calibration: pop_ann × brfss_prev_opt2. | Numeric, count |
| hh_prev_opt3 | Option 3 – Social structure | Estimated proportion of households with at least one bereaved member: 1 − (1 − brfss_prev_opt2)^hh_size_avg. | Numeric probability |
| bereaved_hh_opt3 | Option 3 – Social structure | Estimated bereaved households: hh_total × hh_prev_opt3. | Numeric, count |
| fam_prev_opt3 | Option 3 – Social structure | Estimated proportion of families with at least one bereaved member: 1 − (1 − brfss_prev_opt2)^fam_size_avg. | Numeric probability |
| bereaved_fam_opt3 | Option 3 – Social structure | Estimated bereaved families: fam_total × fam_prev_opt3. | Numeric, count |
| statistical_outlier_flag | Quality assessment | TRUE if the county was flagged by the 1.5×IQR statistical outlier screen on one or more reviewed variables; FALSE otherwise. | Boolean |
| statistical_outlier_vars | Quality assessment | Semicolon-delimited list of variables triggering the 1.5×IQR screen. Blank when statistical_outlier_flag = FALSE. | String |
| logical_outlier_flag | Quality assessment | TRUE if logical inconsistency detected; FALSE otherwise. | Boolean |
| logical_outlier_issues | Quality assessment | Description of logical consistency issue. 'None noted' when logical_outlier_flag = FALSE. | String |

## Intended Use

- Population-level bereavement estimation
- Geographic comparison of mortality environments
- Household and family exposure estimation
- Public health planning and resource allocation
- Methodological sensitivity testing

## Limitations

- Single-state BRFSS baseline probability from Georgia.
- Assumption of parameter transferability from Georgia to Ohio.
- Household and family models assume independence within units.
- ACS margins of error are not propagated into model uncertainty.
- Mortality calibration is based on crude mortality rates, not age-adjusted rates.
- Model outputs represent scenario-based estimates, not direct observations.

## Reconciliation Note

**This README replaces the earlier Supplement 1 language that referenced 2018–2022, Options 1–4, and Option 4 variables.** The current analytic dataset uses the 2017–2018 mortality window, includes Options 1–3 only, and includes quality assessment flags directly in the merged CSV.
