# Supplementary figures and images for: Constructing datasets to measure geographic variation in bereavement: integrating mortality, population structure, and survey-based probability
Source: Front Public Health. 2026 Jul 3;14:1809127. doi: 10.3389/fpubh.2026.1809127 (PMC13378018; doi:10.3389/fpubh.2026.1809127)

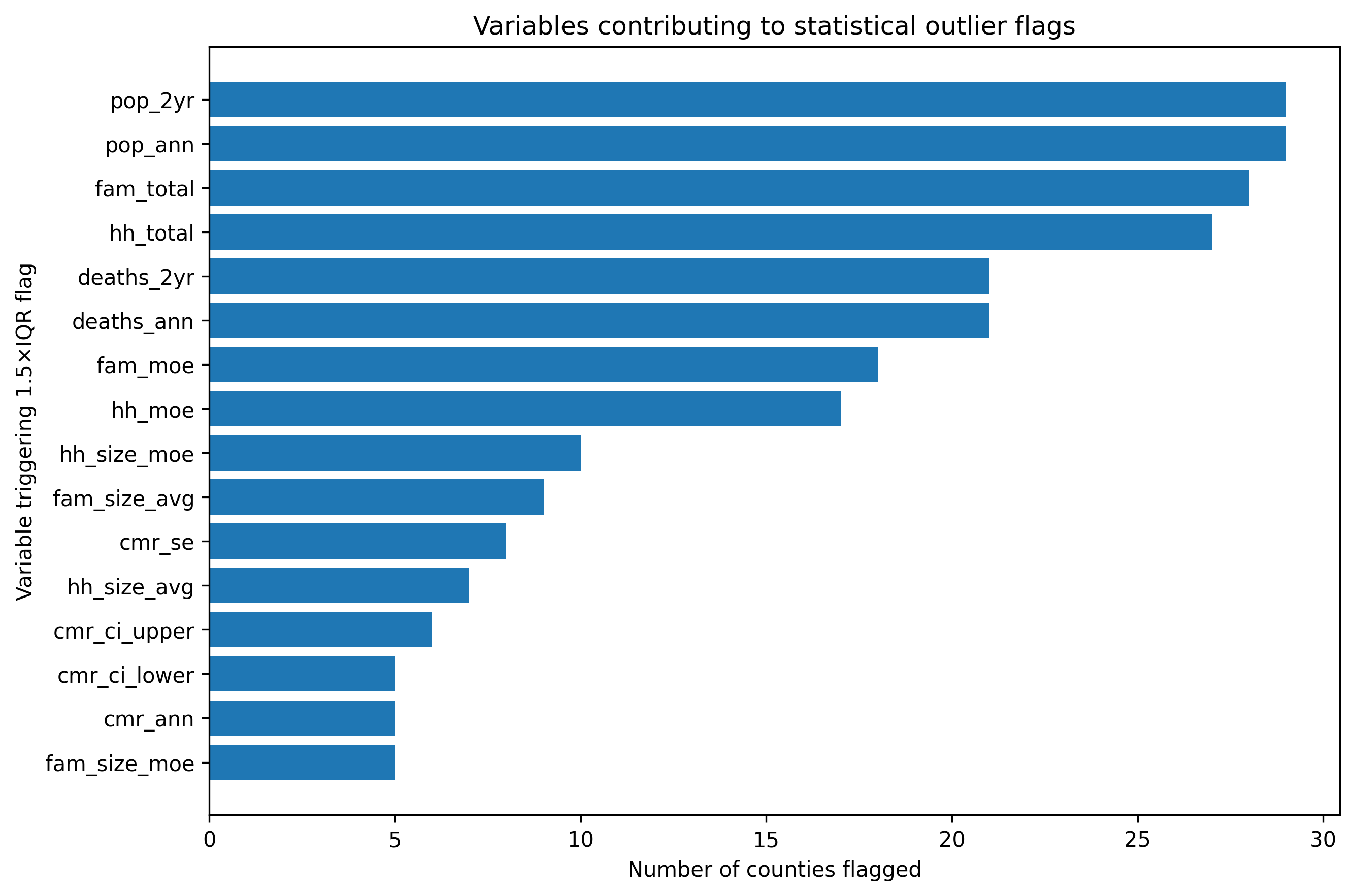

Supplement: Supplement 4 — QA statistical outlier variable frequency may 2. [file Image_1.png]
